# *In vitro* anti-HIV-1 activity of the bioactive compound extracted and purified from two different marine macroalgae (seaweeds) (*Dictyota bartayesiana J.V.Lamouroux* and *Turbinaria decurrens Bory*)

**DOI:** 10.1038/s41598-019-47917-8

**Published:** 2019-08-21

**Authors:** Elumalai Sanniyasi, Gayathri Venkatasubramanian, Madhu Mitra Anbalagan, Preethy P. Raj, Rajesh Kanna Gopal

**Affiliations:** 1 Department of Biotechnology, Rajalakshmi Engineering College, Chennai, Tamil Nadu India; 2 Department of Biotechnology, University of Madras, Guindy Campus, Chennai, Tamil Nadu India; 3 Department of Plant Biology and Plant Biotechnology, Presidency College (Autonomous), Chennai, Tamil Nadu India

**Keywords:** Biotechnology, Biomaterials

## Abstract

Highly active antiretroviral therapy (HAART) is the only available remedial measure to treat HIV infected patients, as recognized by the WHO. However, it is associated with toxicity (nephrotoxicity), high cost and most preferably drug resistance in the first-line treatment. Wherefore, potential and novel natural source is the only option for the modern world to challenge this global issue. In recent years, sulfated polysaccharide from marine macroalgae shown to be biologically active as anti-inflammatory, anticoagulant, antitumor, immunomodulatory and antiviral agents. As a direct inhibitor of HIV including other retroviruses, it is considered as a “new generation antiretroviral drug”. In our present study, Fucoidan, a sulfated polysaccharide has been extracted from two different macroalgae *Dictyota bartayesiana* (DD) and *Turbinaria decurrens* (TD) based on hot water extraction method and further confirmed by FT-IR and RP-HPLC methods. Both the crude and purified fucoidan samples were evaluated for anti-HIV activity after ion exchange chromatography purification. The maximum inhibitory activity of crude and purified fucoidan samples are 90.5% and 89% in the fucoidan extracts of DD. Whereas, it was 89.7% and 92% in the fucoidan extracts of TD. Simultaneously, the IC_50_ values were determined and recorded as 1.56 µg/ml and 57.6 ng/ml in both the crude and purified fucoidan extracts of DD respectively. Similarly, for TD, it was 3 µg/ml and 131.7 ng/ml in the fucoidan extracts of TD. Therefore, further extensive research work is the most needful to fill the gaps to develop this sulfated polysaccharide as a potential drug for the treatment of HIV patients.

## Introduction

Acquired immunodeficiency syndrome (AIDS) is a deadly viral disease that dwells on human immune system has widely dispersed to 36.9 million people around the globe caused by a lentivirus popularly known by HIV (Human Immunodeficiency Virus)^1^. HIV-1 and HIV-2 are two different strains of viral particles consist of similar kind of structure and symptoms, but the latter confined to Africa, whereas the former dispersed to the rest of the world. Highly active antiretroviral therapy (HAART) is the effective treatment for AIDS and recommended by WHO (World Health Organization). Henceforth, toxicity, high production cost, low availability and preferably drug-resistance are the notable factors for the failure rate of the treatment^2^.

For example, Tenofovir is the most frequently used component for HAART recommended by WHO. Concurrently, it has been reported that Tenofovir affects human kidney due to its renal toxicity and causing Fanconi’s syndrome, reduction in the glomerular filtration rate (eGFR) and acute kidney injury (AKI)^3–6^. Hence, less nephrotoxic drugs and simultaneously frequent monitoring of the kidney is preferable for HAART treatment in the Asian Scenario^7^. Liu *et al*.^8^ have reported complex HIV-1 drug resistant mutations in first-line HAART in China and suggested more varieties of drugs for the therapy with timely monitoring of the viral load. Gregson *et al*.^9^ also reported drug resistance in the first-line HAART treatment using tenofovir in the low socio-economic people. This tends us to carry out a research work on the search for some novel anti-HIV therapeutics from the potent natural resources.

Ocean is an enormous source for novel bioactive metabolites which are highly effective and easily available. Marine bioproducts are in huge demand procuring many biological activities including antifungal, antimicrobial, antithrombotic, anti-inflammatory, anticoagulant and antiviral properties^10^. Marine macroalgae or macroalgae are the wonderful gift of Mother Nature with wide range of pharmacological activities. Polysaccharides are the major component of macroalgae and may vary according to the classification of macroalgae such as Chlorophyta (Green algae), Rhodophyta (Red algae) and Phaeophyta (Brown algae). The sulfated polysaccharides have sulphate groups in their molecular structure, have attracted much attention in the field of pharmacology. Intriguingly, brown algae are recorded to accumulate high quantity of sulfated polysaccharides including laminarin, fucoidan and alginates which have anti-HIV activity with wide spectrum of action mechanism^11^. Fucoidan consists of α-L-fucose residue as sole monosaccharide unit of a long chain homopolymer with sulfate groups.

The first most report on the antiviral activity of polysaccharide from seaweed was reported by Gerber *et al*.^12^ in the year 1958, where, the seaweed extracts prevented the chick embryos from the infection of mumps and influenza B viruses. Potentially, effective antiviral property of sulfated polysaccharide from macroalgae was reported against human cytomegalovirus and HSV-1 and HSV-2 (Herpes Simplex Virus)^13^. Since, due to heterogeneity in the structure of sulfated polysaccharide fucoidan, the exact biological reason for their inhibition of retroviral particles is not yet completely derived^14^. However, the sulfated polysaccharide from macroalgae were reported to hamper the replication of retrovirus such as HIV, HSV, respiratory syncytial virus, cytomegalovirus and even dengue virus^15–17^.

It has been reported that the antiviral activity of fucoidan is due to their interaction with the enveloped viral particles or the receptor present on the surface of the host’s cell membrane or may be with the viral enzymes. The sulfated polysaccharide is considered to be rarely tend to alterations by the antigenic drift of the viruses and do not express frequent site of drug-induced resistant mutation. The antiviral activity of sulfated polysaccharide is due to their binding with the HIV particle and hampering the early step of viral infection. The sulfate group may neutralize the positively charged amino acid on the viral envelope glyprotein (gp120) with a strong sulphation motif^18^.

The sulfated polysaccharide is proven to inhibit HIV-1 and HIV-2 along with other enveloped viruses including Herpes Simplex Virus type-1, Influenza virus A and B, Measles virus and respiratory synctial virus. The sulfated polysaccharide inhibits the fusion of virus and the host cell and viral adsorption to the host cells^19^. These virucidal agents have significant potential to inhibit HIV replication^20^. The direct inhibition of HIV-1 replication by the sulfated polysaccharide tends to the availability of new generation potential antiviral drugs^21^. Similarly, the sulfated polysaccharides are antiviral agents against wide spectrum human pathogenic retroviruses by inhibiting the viral replication without toxic effects on the host cells^22^.

In our present investigation, the most commonly found brown macroalgae such as *Turbinaria decurrens* (TD) and *Dictyota bartayesiana* (DD) from the Mandapam Coastal region (Gulf of Mannar) of Rameswaram, Tamil Nadu, India were collected, and evaluated for their bioactive potential for anti-HIV activity.

## Results

### Collection of macroalgae

The marine macroalgae namely *Dictyota bartayesiana* (DD) and *Turbinaria decurrens* (TD) were collected during the month of December 2017 from the Mandapam Coastal region, Rameswaram, Tamil Nadu, India. The collected seaweed samples were washed with water to remove epiphytes, sand particles and dried under shade. The shade dried seaweed biomasses were grounded to fine powder and stored for the extraction of sulfated polysaccharide.

### Extraction and partial purification of sulfated polysaccharide from macroalgae

**Figure 1:**
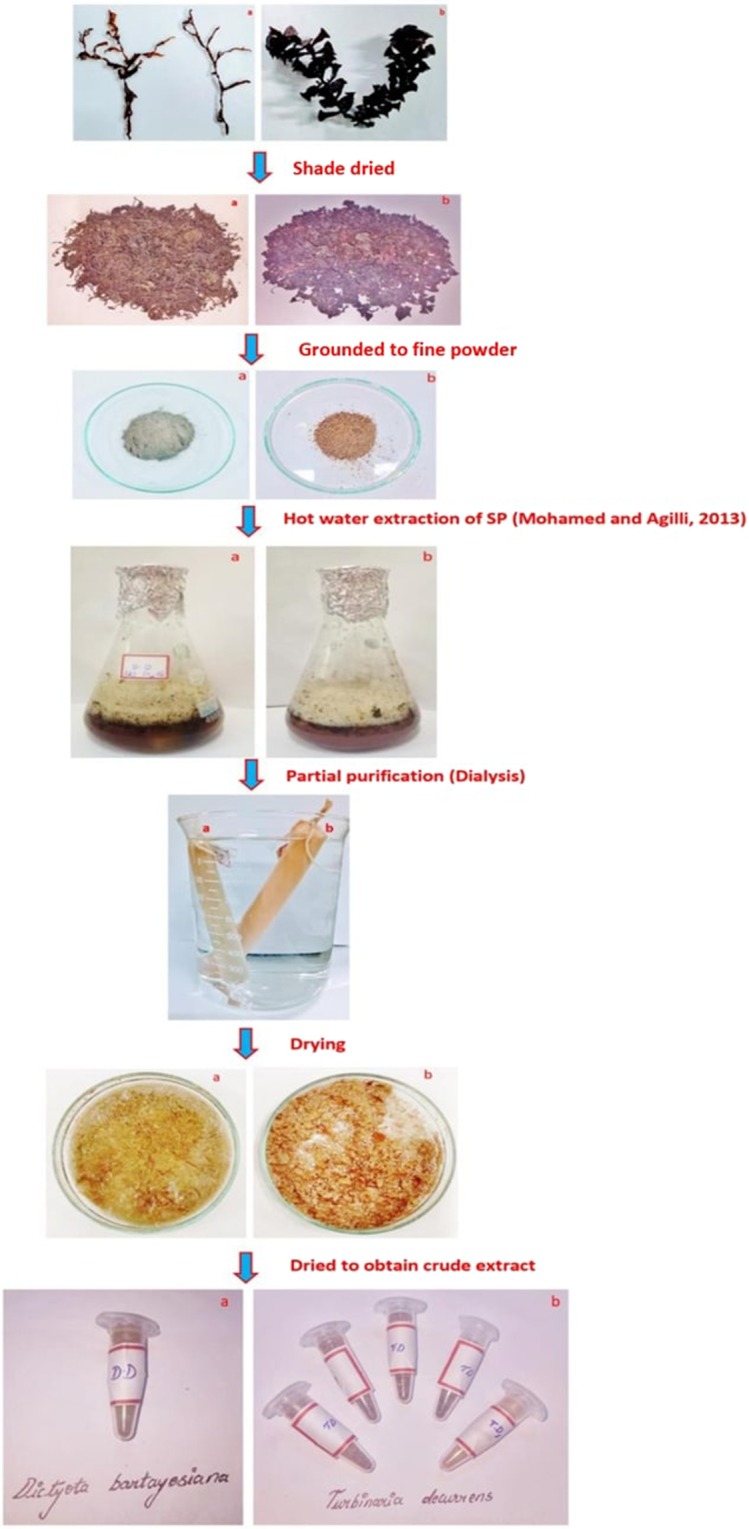
The flow chart showing procedures carried out for the extraction and purification of crude Sulfated Polysaccharide from marine seaweeds (**a**) DD and (**b**) TD.

The shade dried biomasses of two brown macroalgae DD and TD were grounded to fine powder using a mortar and pestle for the extraction of sulfated polysaccharide. Figure 1, showing flow chart exemplifies the methods involved in the extraction and partial purification of crude sulfated polysaccharides. Fine powdered brown algae biomasses in which, DD looks grey ash in color whereas TD looks light brown in color.

The sulfated polysaccharide extracted from brown algae DD and TD were partially purified by dialysis using a cellulose membrane (Fig. 1). Partially purified sulfated polysaccharide of two brown algae DD and TD were subjected to devoid of moisture content by freeze-thaw method to obtain fine powder and yield was calculated. Therefore, the total yield of partially purified sulfated polysaccharide content was 0.768 g and 2.292 g from DD and TD with percentage yield of 7.68% and 22.92% respectively. The crude sulfated polysaccharide was extracted from both the marine macroalgae, and the total sulfated polysaccharide content extracted in DD was comparatively lower when compared to TD.

### Reverse phase - high performance liquid chromatography (RP-HPLC) analysis

The predominant monosaccharide unit found in the sulfated polysaccharide fucoidan is fucose. Hence, in this present study fucose was employed as a standard for RP-HPLC analysis to determine fucoidan content in the two marine seaweed extracts. The standard fucose was purchased from Sigma-Aldrich and the crude sulfated polysaccharide extracts of the two brown algae were predigested with trifluoroacetic acid to hydrolyze fucoidan to yield fucose.

**Figure 2:**
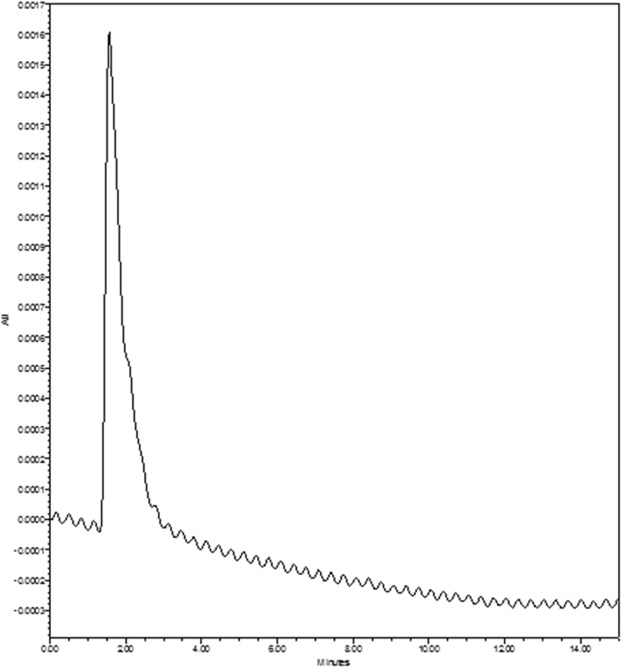
HPLC chromatogram of standard Fucose (Sigma-Aldrich) at RT – 1.56.

**Figure 3:**
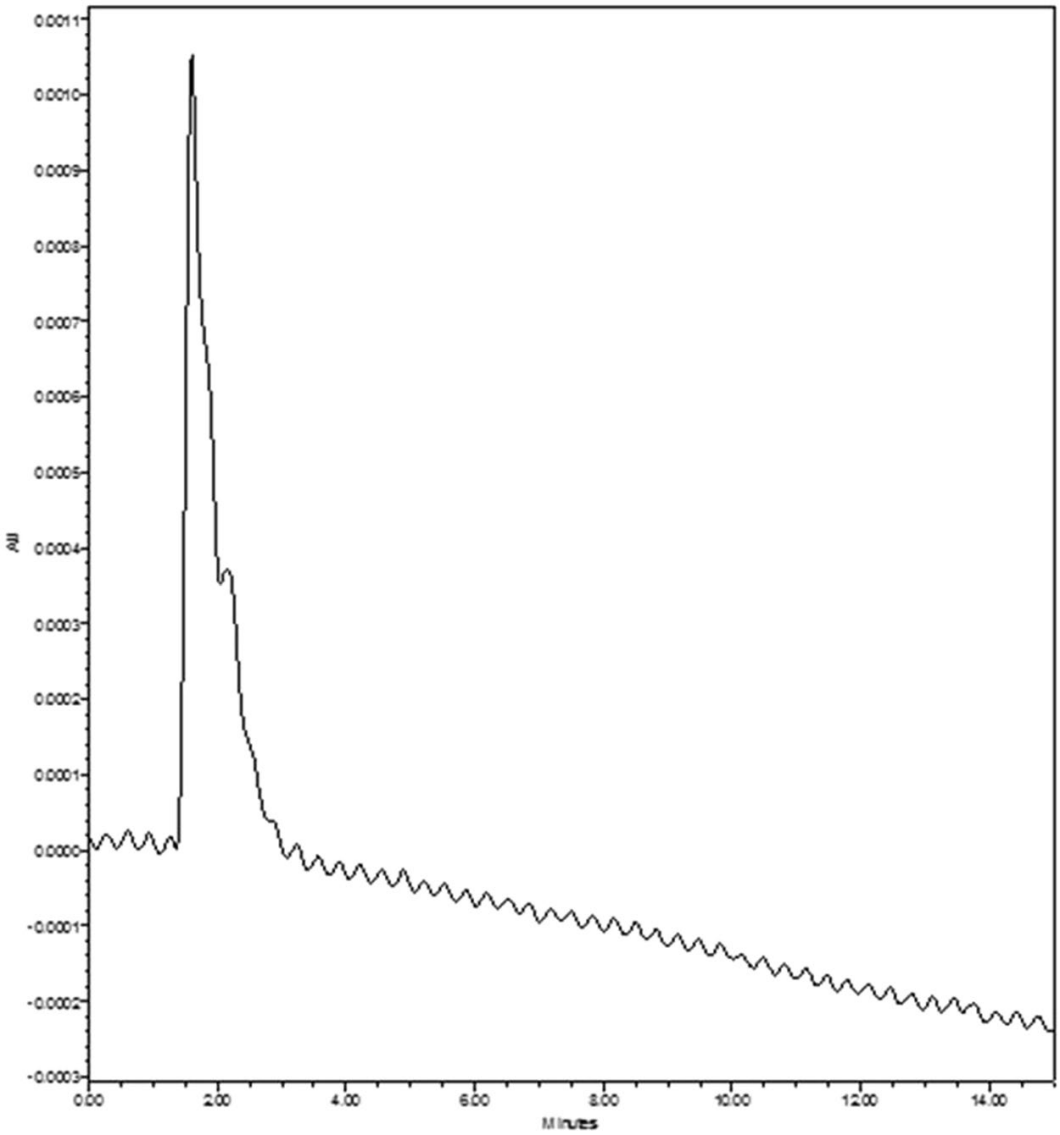
HPLC chromatogram of predigested sulfated polysaccharide extract of *D. bartayesiana* showing Fucose at RT – 1.60.

**Figure 4:**
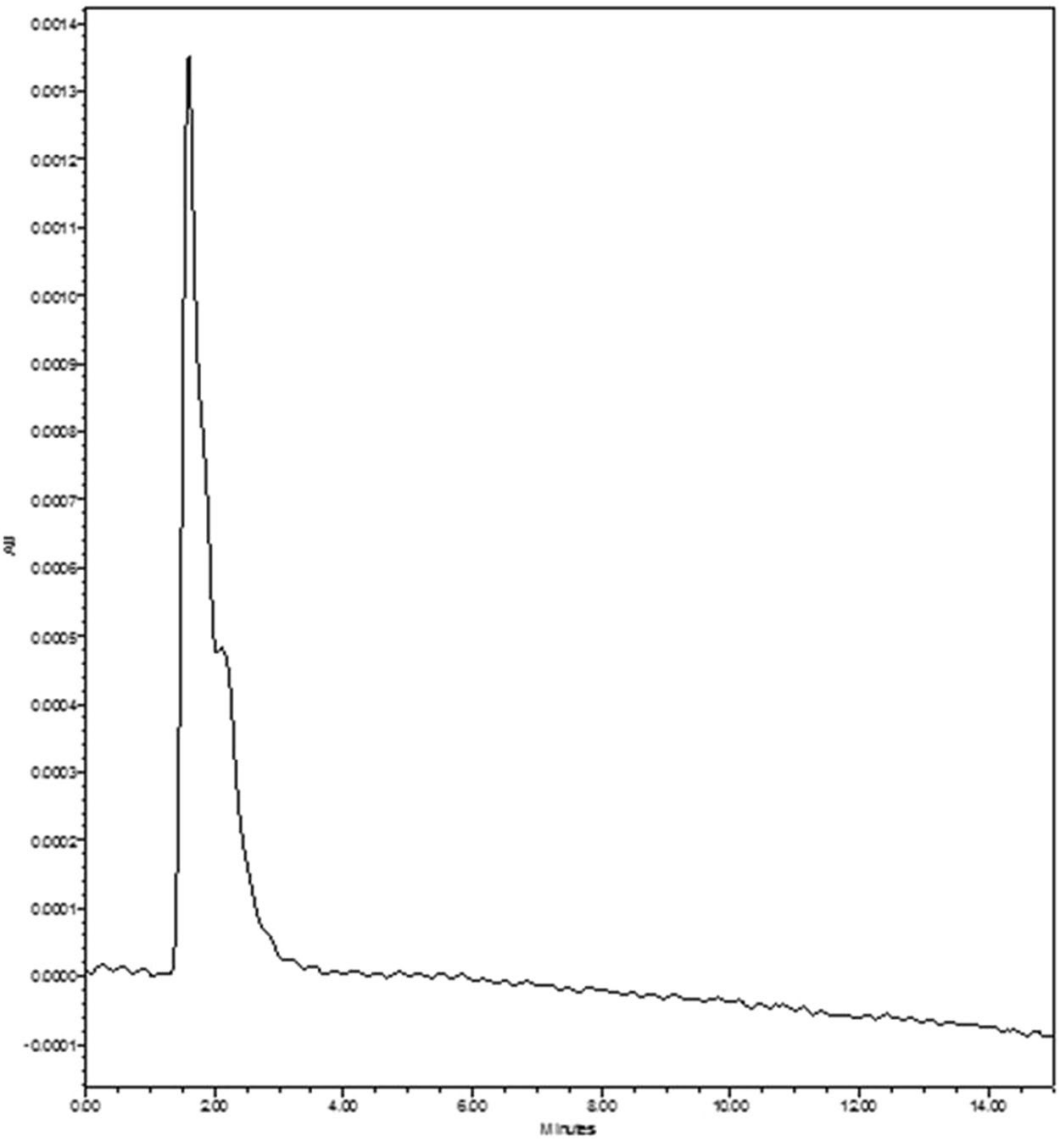
HPLC chromatogram of predigested sulfated polysaccharide extract of *T. decurrens* showing Fucose at RT – 1.59.

Based on the RP-HPLC analysis, the retention time (RT) for standard fucose was 1.56 (Fig. 2). Consequently, the RT for the predigested samples of *D. bartayesiana* and *T. decurrens* were 1.60 and 1.59 respectively (Figs 3 and 4).

**Figure 5:**
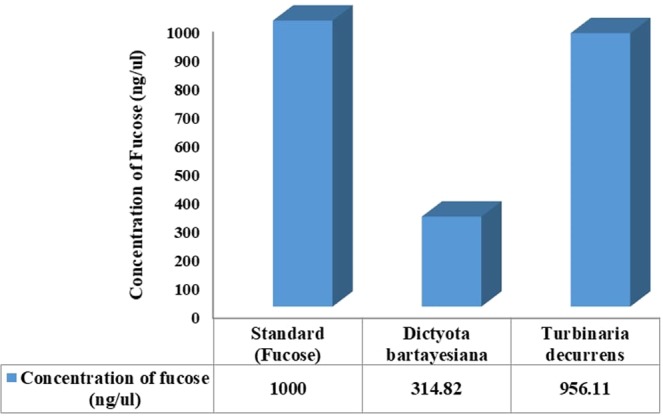
Concentration of fucose present in the seaweed extracts of *D. bartayesiana* and *T. decurrens*.

Similarly, the concentration of fucose content found in the extracts of two brown algae were determined as 314 ng/µl and 956 ng/µl in *D. bartayesiana* and *T. decurrens* respectively (Fig. 5). It was distinctly clear that *T. decurrens* was rich in fucose content than *D. bartayesiana*.

From the overall HPLC results, it is very clear that the fucose content indirectly represents the presence of sulfated polysaccharide, fucoidan and the concentration was comparatively higher in the case of *T. decurrens* than *D. bartayesiana*. Unique peaks were obtained for fucose in both the samples and standard reveals that the higher amount of fucoidan was extracted in both the brown algae *D. bartayesiana* and *T. decurrens*.

### Fourier transform infrared spectrometry (FT-IR)

**Figure 6:**
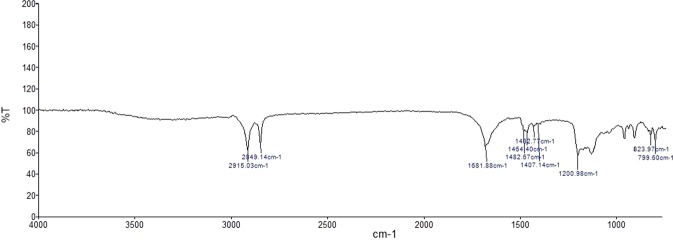
The FT-IR spectrum of crude sulfated polysaccharide of *D. bartayesiana*.

**Figure 7:**
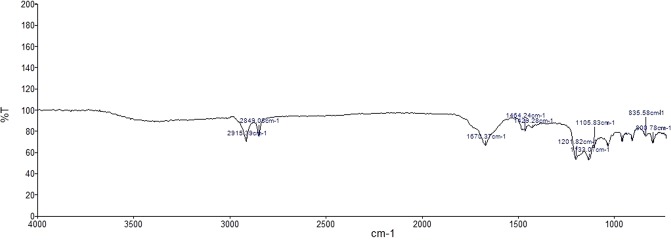
The FT-IR spectrum of crude sulfated polysaccharide of *T. decurrens*.

The occurrence of specific functional groups present in the chemical compounds could be analysed by FT-IR analysis. In this context, the results obtained from FT-IR have shown the occurrence of organic sulphates (R–O–SO_3_^−^) by the presence of down peaks at 1432 cm^−1^ and 1407 cm^−1^ in the crude sulfated polysaccharide of *D. bartayesiana* and 1429 cm^−1^ in *T. decurrens*. Consequently, the peaks at 1200 cm^−1^ and 1201 cm^−1^ in sulfated polysaccharide extracts of *D. bartayesiana* and *T. decurrens* represents the presence of sulphonate groups (R–SO_3_^−^) respectively. Specific peaks at 823 cm^−1^ and 799 cm^−1^ in *D. bartayesiana* and 835 cm^−1^ and 800 cm^−1^ in *T. decurrens* highlights the occurrence of C–O–S stretching of sulphate group in both the sulfated polysaccharide extracts (Tables 1 and 2) (Figs 6 and 7) and other peaks represent the presence of carbohydrate functional groups.

**Table 1:** The FT-IR analysis results showing peaks at the respective wavelength and their respective functional groups of the sulfated polysaccharide extracted from *D. bartayesiana*.

S. No.	Frequency cm^−1^	Functional groups
1	2915	C-H stretch
2	2849	C-H stretch
3	1681	C=O stretch carboxylic group
4	1482	C–C stretch
5	1464	C–H stretch
6	**1432**	**Organic sulfates (R**–**O**–**SO**_**3**_^**−**^**)**
7	**1407**	**Organic sulfates (R**–**O**–**SO**_**3**_^**−**^**)**
8	**1200**	**Sulfonates (R**–**SO**_**3**_^**−**^**)**
9	**823**	**C**–**O**–**S stretching of sulphate group**
10	**799**	**C**–**O**–**S stretching of sulfate group**

**Table 2:** The FT-IR analysis results showing peaks at the respective wavelength and their respective functional groups of the sulfated polysaccharide extracted from *T. decurrens*.

S. No.	Frequency cm^−1^	Functional groups
1	2915	C–H stretch
2	2849	C–H stretch
3	1670	C=O stretch carboxylic group
4	1464	C–H stretch
5	**1429**	**Organic sulfates (R**–**O**–**SO**_**3**_^**−**^**)**
6	**1201**	**Sulfonates (R**–**SO**_**3**_^**−**^**)**
7	**1133**	**Sulfonates (R**–**SO**_**3**_^**−**^**)**
8	**1105**	**Sulfonates (R**–**SO**_**3**_^**−**^**)**
9	**835**	**C**–**O**–**S stretching of sulfate group**
10	**800**	**C**–**O**–**S stretching of sulfate group**

Therefore, based on the results obtained from the RP-HPLC and FT-IR analysis, presence of fucose monosaccharide units and the occurrence of organic sulphate groups in the sulfated polysaccharide extracts of both the brown algae *D. bartayesiana* and *T. decurrens* are thereby confirmed. As a result, the sulfated polysaccharide extracted from both the macroalgae were thereby confirmed as fucoidan.

### Ion exchange chromatography purification of sulfated polysaccharide

**Figure 8:**
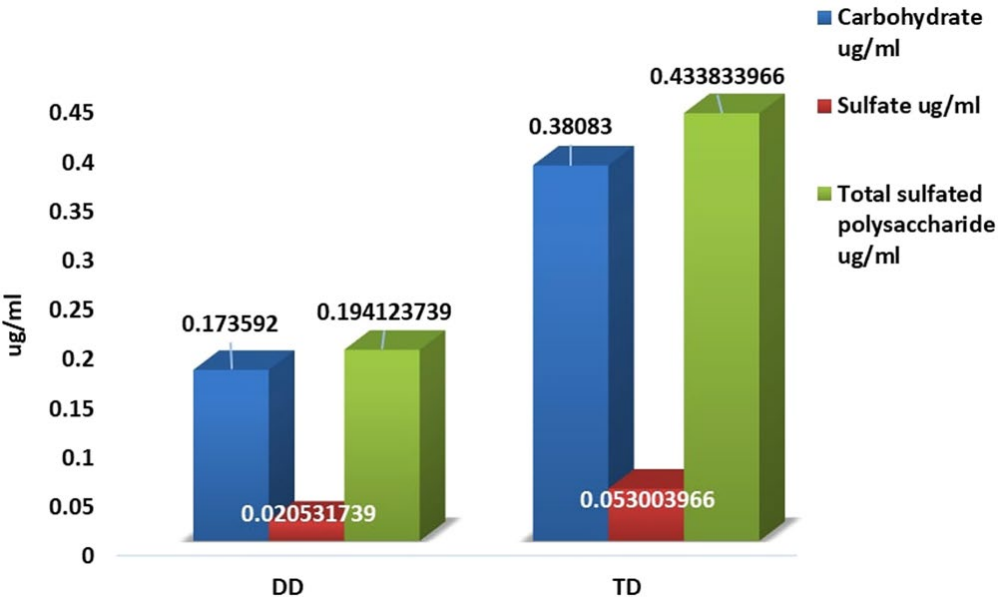
Total sulfated polysaccharide content in DD (*D. bartayesiana*) and TD (*T. decurrens*).

Among the different molar concentrations of NaCl elution, 2.4 M and 0.8 M concentrations of NaCl was found optimum to concentrate sulfated polysaccharide in *D. bartayesiana* and *T. decurrens* respectively (Table 3) and was determined by both the estimation of carbohydrate and sulfate. The total carbohydrate and total sulfate content were 0.173 µg/ml and 0.020 µg/ml in *D. bartayesiana*. Similarly, in *T. decurrens* which were 0.380 µg/ml and 0.053 µg/ml respectively (Fig. 8). The total sulfated polysaccharide content was determined as 0.194 µg/ml and 0.433 µg/ml for *D. bartayesiana* and *T. decurrens* respectively (Fig. 8) and which were about 3.89% and 8.67% of the total biomass.

**Table 3:** The ion exchange purified elution from two different marine macroalgae rich in sulfated polysaccharide.

S. No.	Elution from Ion exchange purification	Total carbohydrate (µg/5 ml)	Total sulphate (µg/5 ml)	Total sulfated polysaccharide (µg/5 ml)
**1**.	***D. bartayesiana*** **(2.4 M NaCl)**	0.86796	0.10625	0.97421
**2**.	***T. decurrens*** **(0.8 M NaCl**	1.90415	0.26502	2.16917

### Anti-HIV activity of crude and purified sulfated polysaccharide extracts

**Figure 9:**
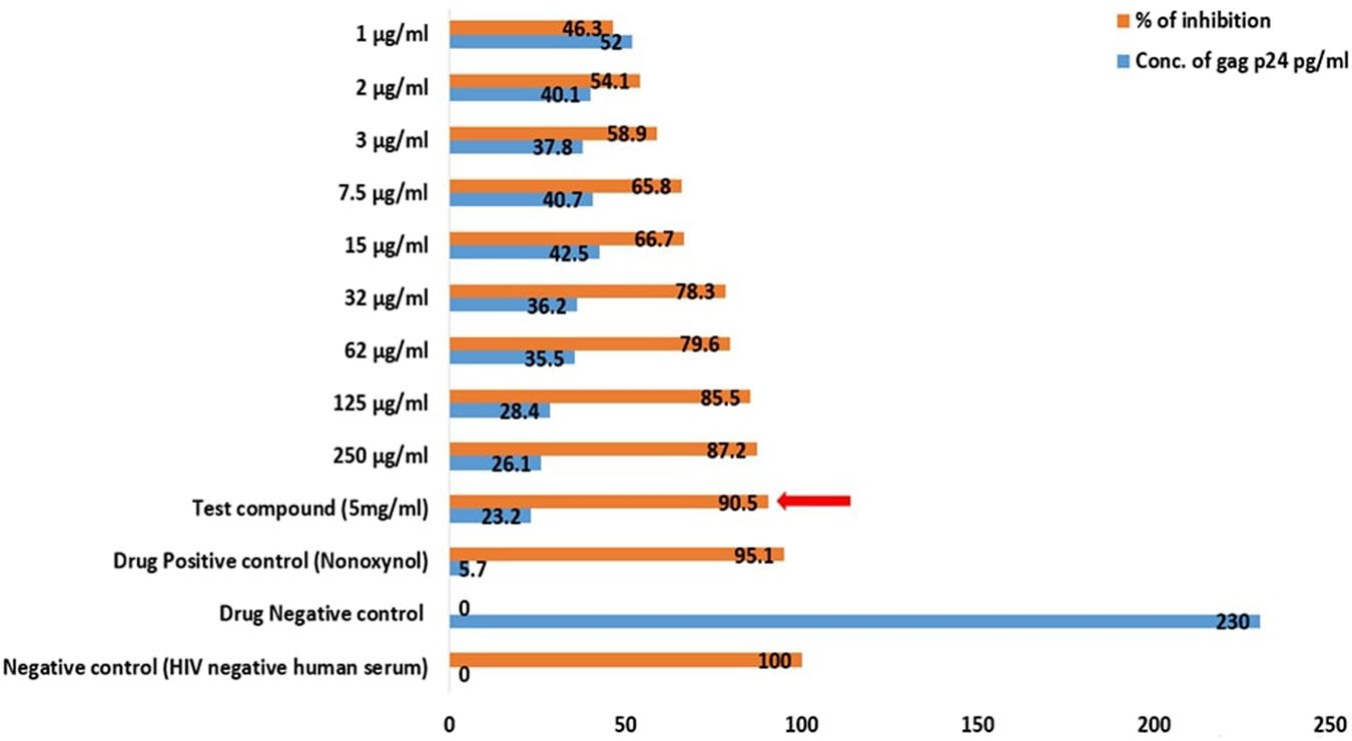
Anti-HIV activity of the crude sulfated polysaccharide extract of *D. bartayesiana* showing the maximum inhibition at its maximum concentration in a red arrow.

**Figure 10:**
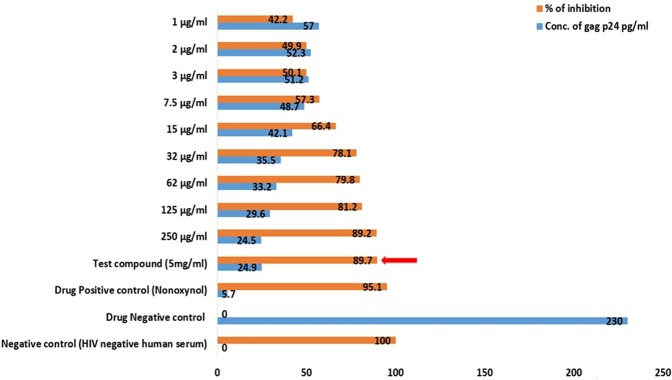
Anti-HIV activity of the crude sulfated polysaccharide extract of *T. decurrens* showing the maximum inhibition at its maximum concentration in a red arrow.

From the results obtained by this assay, it is obviously clear that the inhibition of the HIV proliferation rate was directly proportional to the concentration of the test samples (crude sulfated polysaccharide) of brown algae DD and TD (Figs 9 and 10). The maximum inhibitory activity of crude fucoidan of brown algae DD and TD against HIV were 90.5% and 89.7% at 5 mg/ml concentration respectively. The IC_50_ values measured were 1.56 µg/ml and 3 µg/ml for the crude sulfated polysaccharide extract of DD and TD respectively.

**Figure 11:**
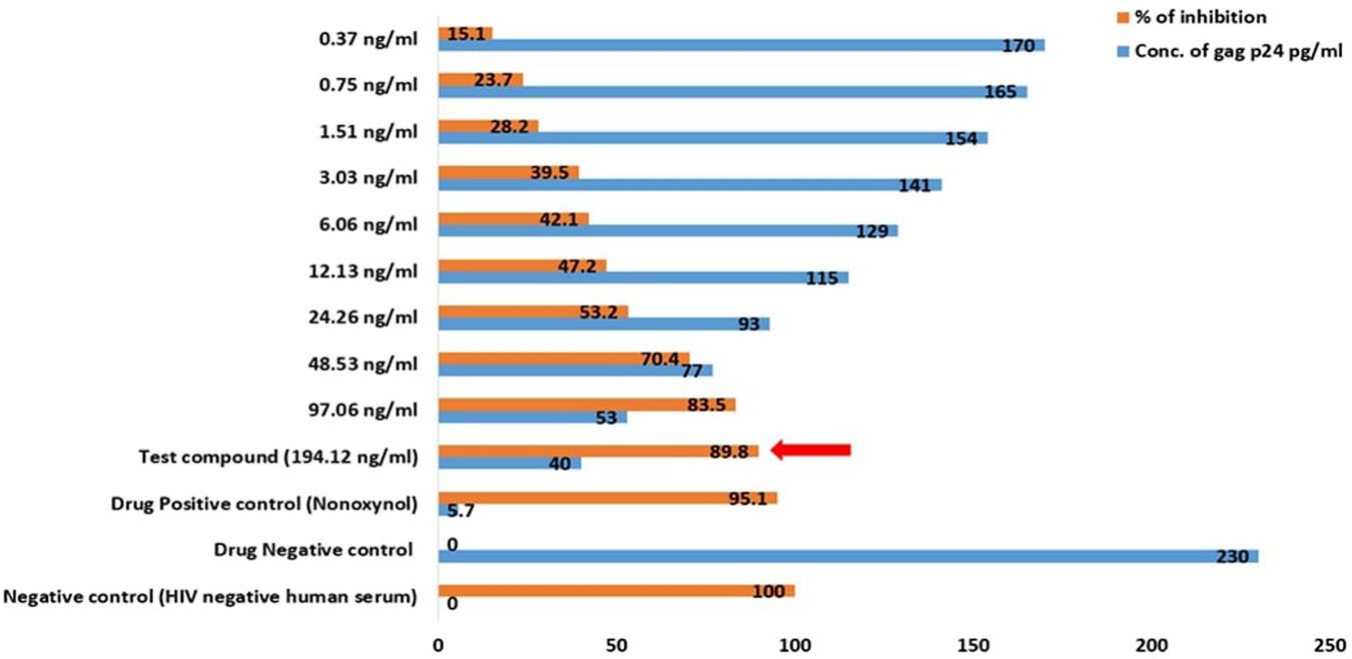
Anti-HIV activity of purified sulfated polysaccharide of the brown alga DD showing the maximum inhibition at its maximum concentration shown in a red arrow.

**Figure 12:**
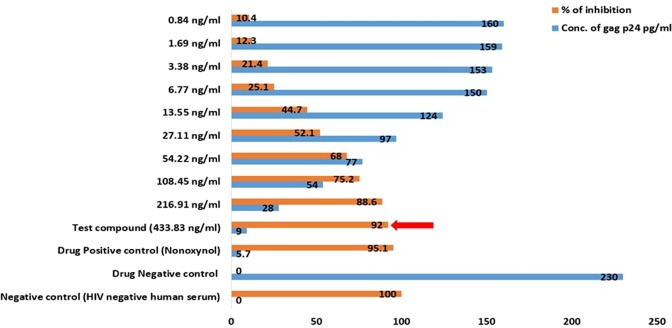
Anti-HIV activity of purified sulfated polysaccharide of TD showing the maximum inhibition at its maximum concentration in a red arrow.

The anti-HIV study was again performed with the purified sulfated polysaccharide samples and the maximum inhibition percentage was found to be 92% and 89% with mean IC_50_ values 131.7 ng/ml and 57.6 ng/ml for TD and DD respectively. And thus, both the purified extracts of DD and TD were found to obtain highly active HIV-inhibitory activity (around 90% inhibitory activity) (Figs 11 and 12).

## Discussion

Seaweed is one among the most valuable, abundant and cheap source of most diverse biologically active compounds. One intimate compound from the seaweed is sulfated polysaccharide which is found associated with the cell walls of the seaweed which gives toughness and flexibility^23^. For example, fucoidan from brown algae (Phaeophyceae), Carrageenan from red algae (Rhodophyceae) and Ulvan from green algae (Chlorophyceae). These diverse sulfated polysaccharides from different algae have different properties due to their uniqueness with respect to their genus or even species levels. Particularly, this organic compound has allured much recognition among scientific researchers pharmaceutically, rather than food and cosmetic industries^24^. Therefore, in our present study, two different macroalgae such as *Dictyota bartayesiana* (DD) and *Turbinaria decurrens* (TD) were collected from the Gulf of Mannar regions.

Due to the heterogeneity of different fucoidan structures from various seaweed sources, different extraction methods were exemplified by various reports^14^. Hot water extraction method discussed by Mohamed and Agilli^25^ was followed in our study. In which, crude polysaccharide extract was partially purified by dialysis and ion exchange chromatography. The crude sulfated polysaccharide extracted was 7.68% and 22.92% from DD and TD respectively, and which was high when compared with the study previously held by Dinesh *et al*.^26^ from *Sargassum swartzii* (5.96%). Recently, many numbers of studies have been reported regarding the antitumor activity^27^, anticoagulant activity^28^, anti-inflammatory activity^29^ and antiviral activity^13^ of fucoidan from different brown algal extracts. Effective immunomodulatory activity of the sulfated polysaccharide fucoidan, have been recorded for anti-metastasis and anti-lymphangiogenesis^30,31^.

Since, antiviral activity of sulfated polysaccharide from macroalgae have been illustrated in many scientific reports. For example, fucan, a complex structure of glucuronic acid and sulfated fucose units extracted from the seaweed *Cladosiphon okamuranus* exhibits anti-dengue activity on type 2 virus (DENV-2)^32^ and other marine seaweed extracts inhibits HSV-1, HSV-2^33–35^. One common feature among these polysaccharides are, the presence of sulfate molecules attached to the sugar molecules. Hidari *et al*.^32^ exemplified that sulphation of fucan, is the most needful for this antiretroviral activity. But, the reduction of carbohydrate molecules into monosaccharide also hampers antiviral property of the compound. Adding to this, sulfated xylomannans extracted from a red alga *Sebdenia polydactyla* inhibits the proliferation of HSV-1 *in vitro* in Vero cells, whereas the antiviral activity of the compound was lost when desulfated and retrieved its potent antiviral activity when over-sulfated^34^.

The HAART involves reverse transcriptase inhibitors as the first-generation ARV drugs licensed and suggested by the WHO, whereas, emerging of drug resistant virulent strains and its side effects are the bottle neck issue^36^. Wherefore, search for novel potent bioactive compounds from natural resources is the most needful research for antiretroviral therapy^37^. Our study is also one of the solutions to this issue by evaluating the bioactivity of sulfated polysaccharide extracted from two different macroalgae DD and TD from the Gulf of Mannar region for its antiretroviral activity.

The FT-IR results confirmed the presence of sulfate content in the crude extracts of DD and TD by the occurrence of organic sulfate groups which coincides with the results reported previously by Sinha *et al*.^38^. Similarly, the occurrence of fucoidan was hereby confirmed by RP-HPLC method with standard L-fucose as reference in both the crude SP extracts. From the purified crude extract, the total sulfated polysaccharide content was estimated as 3.89% and 8.67% with respect to DD and TD.

The fucoidan inhibits the infection of HIV to the host cell surface and hence effective in virucidal activity^34^. However, *in vitro* inhibition of HIV through prevention of viral replication was reported from the sulfated polysaccharides extracted from two red macroalgae^21^ at an IC_50_ value of 12.5 µg/mL and HSV-1 at 4.1 µg/mL and 17.2 µg/mL. Concurrently, the crude fucoidan extracts from DD and TD in this present study were subjected to the evaluation of anti-HIV activity and found inhibiting the propagation of HIV at an IC_50_ value of 1.56 µg/ml and 3 µg/ml respectively. Similarly, the average anti-HIV and IC_50_ value of three brown seaweed extracts including *Sargassum polycystum*, *S. mcclurei* and *Turbinaria ornata* were between 0.33 and 0.7 μg/ml^18^, which is low when compared with the present study.

Thuy *et al*.^18^ also concluded that the fucoidan was effective when pre-incubated with the virus rather incubated with the host cells and was ineffective after infection. However, the anti-HIV and IC_50_ value of sulfated polysaccharide extracts from macroalgae *E. arborea* and *S. filiformis* resp. were 0.275 𝜇g/ml and 0.985 𝜇g/ml^39^. Comparatively, Prokofjeva *et al*.^40^ suggested that high molecular weight fucoidans from *Saccharina cichorioides* and *S. japonica* were the most effective retroviral inhibitors. As a result, all the natural source of fucoidans are considered to be a potent anti-HIV compounds with irrespective of their sugar backbone and degree of sulfation even at very low concentrations from 0.001–0.05 μg/ml. Therefore, the IC_50_ value of the purified fucoidan extracts of TD and DD from our present investigation were 131.7 ng/ml and 57.6 ng/ml and inhibits about 92% and 89% at maximum concentration with highly active HIV-inhibitory activity.

## Methods

### Collection of macroalgae

Diversely, macroalgae rich, Mandapam Coastal region (East Coast) from Rameswaram, Tamil Nadu, India was chosen for the collection of macroalgae samples to carry out our present study. Brown macroalgae was given importance due to the bioavailability of sulfated polysaccharide, and hence two different macroalgae *Turbinaria decurrens J.V.Lamouroux* (TD) and *Dictyota bartayesiana Bory* (DD) belonging to the family Phaeophyceae were collected from the above-mentioned site.

### Extraction and partial purification of sulfated polysaccharide from macroalgae

The extraction of sulfated polysaccharide (SP) from the two different macroalgae (TD and DD) was carried out based on the hot water extraction method described by Mohamed and Agilli^25^. About 10 g of finely grounded seaweed biomasses were taken individually for depigmentation using petroleum ether and incubated overnight, followed by acetone in the second phase. The depigmented and air-dried residual seaweed powders were subjected to acid treatment with 0.1 M HCl at room temperature under constant stirring for 1 hour and kept for overnight incubation, followed by alkali treatment with 2% Na_2_CO_3_ at 90 °C for 2 hours.

The residual contents were subjected to hot water extraction at 90 °C for 2 hours and repeated twice. After hot water extraction, the whole content was filtered through Whatman No.1 filter paper. The filtrate was then treated with 1 g of CTAB (Cetyltrimethyl ammonium bromide) for 15 min., the obtained precipitate was filtered through Whatman No.1 filter paper, washed twice with Milli-Q water and followed by the treatment with 20% ethanolic potassium iodide (KI) (20 g of KI in 80 ml of water in 20 ml of ethanol) for 30 min. The resultant precipitate was again washed twice with Milli-Q water and stored in a refrigerator at 4 °C. Then, the obtained crude sulfated polysaccharide extracts from both the macroalgae were partially purified by dialysis using Dialysis membrane-70 from HiMedia against Milli-Q water at 15 °C for two days. Then the residual contents were subjected to drying by freeze-thaw method intermittently to obtain dry powder, weighed and stored for further processes.

### Reverse phase - high performance liquid chromatography (RP-HPLC) analysis

Prior to RP-HPLC analysis, the crude sulfated polysaccharide extracts were hydrolyzed with trifluoroacetic acid (TFA). About 10 mg of crude sulfated polysaccharide extracts of both the macroalgae were predigested with 2 ml of 2 M TFA at 121 °C for 1 hour. Then after hydrolysis, the reaction medium was dried with vacuum concentrator and dissolved using distilled water. Then the resultant solutions were neutralized at pH 7.0 with 1 N NaOH^41^.

The conditions for RP-HPLC are HPLC pump with C18 column, Water 2489 UV/Vis – 274 nm detector, HPLC grade water as mobile phase at flow rate of 0.6 ml/min. with injection volume of 1 ml. The concentration of samples were 1 µg/µl and same for standard L-Fucose obtained from Sigma-Aldrich.

### Fourier transform infrared spectrometry (FT-IR)

About 1 mg of each crude sulfated polysaccharide extracts were mixed with 100 mg of potassium bromide (KBr) in a mortar and pestle separately and placed in a pellet die to compress up to 10,000 psi. Then, the obtained pellet was placed in the FT-IR sample holder for analysis. The samples were analyzed by FT-IR spectrometer in a frequency range between 600 and 4000 cm^−1^.

### Ion exchange purification of sulfated polysaccharide

The crude sulfated polysaccharide extracts of both the brown algae were further purified using anion exchange chromatography (DEAE (Diethylaminoethyl cellulose) Sepharose from Sigma-Aldrich, 15 × 2.5 cm column). The samples were eluted with 5 ml of different molar concentrations of NaCl ranging from 0 to 3.6 M with 0.4 M interval in 50 mM sodium acetate buffer with pH 5.0. Each fraction was collected, dialyzed (Dialysis membrane-70, HiMedia) against Milli-Q water to free the fractions from NaCl and conserved for further processes.

### Determination of carbohydrate and sulfate content

The total carbohydrate and sulfate content were determined in all the fractions after ion exchange purification based on the Phenol sulphuric acid method^42^ and sodium rhodizonate method^43^ respectively.

### Anti-HIV activity of crude and purified sulfated polysaccharide extract

For the determination of anti-HIV activity of crude and purified sulfated polysaccharide extract, HIV-1 (Clade C strain) was obtained from the National Aids Research Institute (NARI, Pune, India) and incubated on human PBMC (Peripheral blood mononuclear cells) as described by Jackson *et al*.^44^. HIV Gag p24 assay was performed on blasted PBMC for the determination of anti-HIV activity of the test compound^45^. All the assays were performed in the biosafety level 2^+^ (BSL). The test compounds and a positive control (Nonoxynol-9) were treated in a series of concentrations with 15 pg of HIV-1 virus and incubated for 1 hour at 37 °C. Then after incubation, blasted PBMC cells (0.3 × 10^6^ concentrations) was added and incubated for 37 °C for 2 hours. The cells were washed and re-suspended in 2% RPMI (Rosewell Park Memorial Institute) medium and further incubated for 5 days at 37 °C.

Three different groups were segregated namely the drug-positive control group (Nonoxynol-9), drug-negative control group (treated with distilled water) and a test compound group. After five days of incubation, the supernatants were collected and analyzed for anti-HIV activity by HIV Gag p24 protein concentration using ELISA (Enzyme-linked immune sorbent assay) plate assay^46^. The absorbance values were measured at 450 nm by ELISA plate reader, inhibitory concentrations values were determined for the respective concentrations of the test compound and the results were interpreted.

## Conclusion

Drug resistance and toxicity are the associated issues with HAART while treating HIV in patients. Hence, search for a novel and potent natural source is an audible solution for this major issue in the upcoming decades. As a result, from our present study, fucoidan is one among the potent natural sulfated polysaccharide extracted from two different macroalgae *Dictyota bartayesiana* (DD) and *Turbinaria decurrens* (TD) and evaluated for its anti-HIV activity. Fucoidan was extracted based on hot water extraction method and further confirmed based on FT-IR and RP-HPLC methods. Both the crude fucoidan and purified fucoidan samples were evaluated for anti-HIV activity after ion exchange chromatography purification. The maximum inhibitory activity of crude and purified fucoidan samples are 90.5% and 89% in the fucoidan extracts of DD. Whereas, it was 89.7% and 92% in the fucoidan extracts of TD. Simultaneously, the IC_50_ values were determined and recorded as 1.56 µg/ml and 57.6 ng/ml in both the crude and purified fucoidan extracts of DD respectively. Similarly, for TD, it was 3 µg/ml and 131.7 ng/ml in the fucoidan extracts of TD. Furthermore, extensive research work is essential for developing this bioactive compound for the clinical treatment of HIV in patients.

## Data Availability

All the data and supporting materials are available with the corresponding author.

## References

[1] UNAIDS. Fact sheet –World AIDS Day 2018. 2030. *Ending the AIDS Epidemic*. 1–6 (2017).

[2] Artan M, Karadeniz F, Karagozlu MZ, Kim M-M, Kim S-K (2010). Anti-HIV activity of low molecular weight sulfated chitooligosacharides. Carbohydrate Research..

[3] Hamzah L (2017). Treatment‐limiting renal tubulopathy in patients treated with tenofovir disoproxil fumarate. J. Infect..

[4] Suzuki S (2017). Effect of tenofovir disoproxil fumarate on incidence of chronic kidney disease and rate of estimated glomerular filtration rate decrement in HIV‐1‐infected treatment‐naïve Asian patients: results from 12‐year observational cohort. AIDS Patient Care STDS..

[5] Tanuma J (2016). Renal dysfunction during tenofovir use in a regional cohort of HIV‐infected individuals in the Asia‐Pacific. PLoS One..

[6] Zuniga M (2017). Tenofovir‐associated Fanconi’s syndrome and rickets in a HIV infected girl. Rev. Chil. Pediatr..

[7] Joshi K (2018). Changes in renal function with long‐term exposure to antiretroviral therapy in HIV‐infected adults in Asia. Pharmacoepidemiol Drug Saf..

[8] Liu J (2015). Population -based human immunodeficiency virus 1 drug resistance profiles among individuals who experienced virological failure to first-line antiretroviral therapy in Henan, China during 2010–2011. AIDS Res. Ther..

[9] Gregson J, Ndembi N, Hamers RL, Marconi VC (2016). Global epidemiology of drug resistance after failure of WHO recommended first-line regimens for adult HIV-1 infection: a multicenter retrospective cohort study. The Lancet Infectious Diseases..

[10] Rabanal M, Ponce NMA, Navarro DA, Gomez RM, Strotz CA (2014). The system of fucoidans from the brown seaweed *Dictyota dichotoma*: Chemical analysis and antiviral activity. Carbohydrate Polymers..

[11] Lee JB (2006). Antiviral sulfated polysaccharide from Navicula directa, a diatom collected from deep-sea water in Toyama bay. Biol. Pharm. Bull..

[12] Gerber P, Dutcher JD, Adams EV, Sherman JH (1958). Protective effect of seaweed extracts for chicken embryos infected with influenza B or mumps virus. Proc. Soc. Exp. Biol. Med..

[13] Jung-Bum L, Hayashi K, Hashimoto M, Nakano T, Hayashi T (2004). Novel antiviral fucoidan from Sporophyll of *Undaria pinnatifida* (Mekabu). Chem. Pharm. Bull..

[14] Jiao G, Yu G, Zhang J, Ewart HS (2011). Chemical structures and bioactivities of sulfated polysaccharides from marine algae. Marine Drugs..

[15] Damonte EB, Matulewicz MC, Cerezo AS (2004). Sulfated seaweed polysaccharides as antiviral agents. Curr. Med. Chem..

[16] Luescher-Mattli M (2003). Algae, a possible source for new drugs in the treatment of HIV and other viral diseases. Curr. Med. Chem..

[17] Schaeffer DJ, Krylov VS (2000). Anti-HIV activity of extracts and compounds from algae and cyanobacteria. Ecotoxicol. Environ. Saf..

[18] Thuy TTT (2015). Anti-HIV activity of fucoidans from three brown seaweed species. Carbohydrate Polymers..

[19] Esteves AIS, Nicolai M, Humanes M, Goncalves J (2011). Sulfated polysaccharides in marine sponges: extraction and anti-HIV activity. Marine Drugs..

[20] Hashimoto K (1996). Antiviral activity of a sulfated polysaccharide extracted from the marine Pseudomonas and marine plant Dinoflagellata against human immunodeficiency viruses and other enveloped viruses. Antiviral Chemistry and Chemotherapy..

[21] Bouhlal R (2011). Antiviral activities of sulfated polysaccharides isolated from *Sphaerococcus coronopifolius* (Rhodophyta, Gigartinales) and *Boergeseniella thuyoides* (Rhodophyta, Ceramiales). Marine Drugs..

[22] Haslin C, Lahaye M, Pellegrini M, Chermann JC (2001). *In vitro* anti-HIV activity of sulfated cell-wall polysaccharides from gametic, carposporic and tetrasporic stages of the Mediterranean red alga *Asparagopsis armata*. Planta Med..

[23] Wijesinghe WAJP, Jeon Y (2012). Biological activities and potential industrial applications of fucose rich sulfated polysaccharides and fucoidans isolated from brown seaweeds: A review. Carbohydr. Polym..

[24] Cunha L, Grenha A (2016). Sulfated seaweed polysaccharides as multifunctional materials in drug delivery applications. Marine Drugs..

[25] Mohamed SF, Agili FA (2013). Antiviral sulfated polysaccharide from brown algae *Padina pavonia* characterization and structure elucidation. International Journal of Chem. Tech. Research..

[26] Dinesh S (2016). *In vitro* anti-HIV-1 activity of fucoidan from *Sargassum swartzii*. International Journal of Biological Macromolecules..

[27] Kwak J (2014). Fucoidan as a marine anticancer agent in preclinical development. Marine Drugs..

[28] Taylor P, Nishino T, Aizu Y, Nagumo T (1991). The relationship between the molecular weight and the anticoagulant activity of two types of fucan sulfates from the brown seaweed *Ecklonia kurome*. Agric. Biol. Chem..

[29] Young H (2011). Anti-inflammatory effects of fucoidan through inhibition of NF-B, MAPK and Akt activation in lipopolysaccharide-induced BV2 microglia cells. Food Chem. Toxicol..

[30] Raghavendran HRB, Srinivasan P, Rekha S (2011). Immunomodulatory activity of fucoidan against aspirin-induced gastric mucosal damage in rats. Int. Immunopharmacol..

[31] Teng H (2015). Fucoidan suppresses hypoxia-induced lymphangiogenesis and lymphatic metastasis in mouse hepatocarcinoma. Marine Drugs..

[32] Hidari KIPJ (2008). Structure and anti-dengue virus activity of sulfated polysaccharide from a marine alga. Biochem. Biophys. Res. Commun..

[33] Adhikari U (2006). Structure and antiviral activity of sulfated fucans from *Stoechospermum marginatum*. Phytochemistry..

[34] Ghosh T (2009). Focus on antivirally active sulfated polysaccharides: From structure-activity analysis to clinical evaluation. Glycobiology..

[35] Harden EA, Falshaw R, Carnachan SM, Kern ER, Prichard MN (2009). Virucidal activity of polysaccharide extracts from four algal species against herpes simplex virus. Antiviral Res..

[36] Bangsberg DR (2000). Adherence to protease inhibitors, HIV-1 viral load, and development of drug resistance in an indigent population. AIDS..

[37] Olisah VO, Baiyewu O, Sheikh TL (2010). Adherence to highly active antiretroviral therapy in depressed patients with HIV/AIDS attending a Nigerian University teaching hospital clinic. African Journal of Psychiatry..

[38] Sinha S, Astani A, Ghosh T, Schnitzler P, Ray B (2010). Polysaccharides from *Sargassum tenerrimum*: Structural features, chemical modification and anti-viral activity. Phytochemistry..

[39] Morán-Santibañez Karla, Cruz-Suárez Lucia Elizabeth, Ricque-Marie Denis, Robledo Daniel, Freile-Pelegrín Yolanda, Peña-Hernández Mario A., Rodríguez-Padilla Cristina, Trejo-Avila Laura M. (2016). Synergistic Effects of Sulfated Polysaccharides from Mexican Seaweeds against Measles Virus. BioMed Research International.

[40] Prokofjeva MM (2013). Fucoidans as potential inhibitors of HIV-1. Marine Drugs..

[41] Chen Xiaolin, Yang Shengfeng, Wang Jinxia, Song Lin, Xing Ronge, Liu Song, Yu Huahua, Li Pengcheng (2015). Sulfated Polysaccharides Isolated from ClonedGrateloupia filicinaand Their Anticoagulant Activity. BioMed Research International.

[42] Dubois M, Gilles KA, Hamilton JK, Rebers PA, Smith F (1956). Colorimetric method for determination of sugars and related substances. Anal. Chem..

[43] Terho TT, Hartiala K (1971). Method for determination of the sulfate content of glycosaminoglycans. Analytical Biochemistry..

[44] Jackson JB, Erice A, Englund JA, Edson JR, Balfour HH (1998). Prevalence of cytomegalovirus antibody in hemophiliacs and homosexuals infected with human immunodeficiency virus type 1. Transfusion..

[45] Wang R-R (2008). Anti-HIV1 activities of compounds isolated from the medicinal plant *Rhus chinensis*. Journal of Ethnopharmacology..

[46] Pasetto S, Pardi V, Murata RM (2014). Anti-HIV-1 Activity of Flavonoid Myricetin on HIV-1 Infection in a Dual-Chamber *in Vitro* Model. Plos One..

